# Progress towards elimination of onchocerciasis transmission in Mali: A “pre-stop MDA” survey in 18 transmission zones

**DOI:** 10.1371/journal.pntd.0011632

**Published:** 2023-11-15

**Authors:** Housseini Dolo, Michel Emmanuel Coulibaly, Moussa Sow, Yaya Ibrahim Coulibaly, Mama Doumbia, Moussa Sangare, Abdoul Sanogo, Benoit Dembele, Boubacar Guindo, Mamadou Coulibaly, Modibo Keita, Lamine Soumaoro, Dansine Diarra, Boubacar Dicko, Louise Hamill, Seydou Doumbia, Hamadoun Sangho, Yacouba Sangare, Yaobi Zhang, Jamie Tallant, Alpha Seydou Yaro, Charles Mackenzie, Thomas B. Nutman, Daniel Boakye

**Affiliations:** 1 Neglected Tropical Diseases Research Unit, Faculty of Medicine and OdontoStomatology, USTTB, Bamako, Mali; 2 Onchocerciasis Control Programme, National Health and Public Hygiene, Bamako, Mali; 3 Helen Keller International, Bamako, Mali and Dakar, Senegal; 4 SightSavers International, Mali Country Office, Bamako, Mali, and UK Office, Haywards Heath, England, United Kingdom; 5 Faculty of History and Geography, University of Social Sciences and Management of Bamako, Bamako, Mali; 6 Faculty of Medicine and OdontoStomatology, USTTB, Bamako, Mali; 7 Helen Keller International, New York, New York State, United States of America; 8 The END Fund, Neglected Tropical Diseases, New York, New York State, United States of America; 9 Faculty of Technical Sciences of Mali, USTTB, Bamako, Mali; 10 COR-NTD, Task Force for Global Health, Atlanta, Georgia, United States of America; 11 Laboratory of Parasitic Diseases, NIH, Bethesda, Maryland, United States of America; 12 Noguchi Memorial Institute for Medical Research, University of Ghana, Legon, Accra, Ghana; Washington University School of Medicine, UNITED STATES

## Abstract

**Background:**

Onchocerciasis control activities in Mali began in 1975 with vector larviciding carried out by the Onchocerciasis Control Programme (OCP), followed by the distribution of ivermectin from 1998 until the closure of the OCP in 2002. At that time, epidemiological evaluations, using skin snip microscopy and O-150 pool screening PCR in black flies, indicated that the disease had been largely controlled as a public health problem. Ivermectin distribution was nevertheless continued after 2002 in 34 of the 75 health districts in Mali as these were known to still be meso- or hyper-endemic for onchocerciasis. In addition, the onchocerciasis sites known to be hypo-endemic for onchocerciasis benefited from the distribution of ivermectin treatment as part of the mass drug administration (MDA) program for lymphatic filariasis. Various entomological and epidemiological evaluations have now indicated that Mali may have achieved successful interruption of onchocerciasis transmission.

**Methods:**

A series of cross-sectional surveys to update vector breeding sites throughout the endemic areas, followed by a pre-stop ivermectin mass drug administration (Pre-stop MDA) survey, were undertaken in 2019–2020. Based on breeding site findings, historical epidemiological assessments, and vector collection site maps, 18 operational transmission zones (OTZ) were delineated within which a total of 104 first line villages were selected for evaluation. Dried blood spots (DBS) samples were collected from 10,400 children (5–9 years old) from these 104 first line villages and processed for the presence of OV16 antibody using a lab-based rapid diagnostic test.

**Results:**

Within the 544 *Simulium damnosum s*.*l*. breeding sites visited in all five endemic onchocerciasis endemic regions of Mali 18.01% (98/544) were seen to be active with the presence of at least one stage of *S*. *damnosum*. The overall prevalence of OV16 positive children was 0.45% (47/10,400). However, two hotspots were identified: 2.60% (13/500) seroprevalence in the OTZ number 5 in Kayes Region and 1.40% (7/500) in the OTZ number 1 of Sikasso Region.

**Conclusion:**

These data show that onchocerciasis prevalence in the five endemic regions has declined to levels that indicate that Stop-MDA surveys should be now carried out in most of the OTZ except for one in the Kayes Region. This latter site will need additional ivermectin treatment before reevaluation, and an OTZ in the Sikasso Region requires revaluation before possibly reinitiating MDA.

## Author summary

The control of onchocerciasis, then an endemic disease in Mali, began through vector control by spraying insecticides in *Simulium damnosum s*.*l*. breeding sites in an effort known as the Onchocerciasis Control Program in West Africa (OCP). As the OCP’s activities began to wind down, control activities moved to a combination of vector control together with the distribution of the anthelminthic, ivermectin administered to those populations at risk of infection in the endemic areas. This drug administration was carried out by mobile teams using the framework of community-directed ivermectin treatment (CDTI). It is also important to note that lymphatic filariasis is endemic in all health districts, and thus the onchocerciasis endemic districts, regardless of endemicity, all therefore also benefited from the effects of the additional ivermectin treatment.

After various evaluations of endemicity it was found that Mali qualified to conduct pre-stop ivermectin treatment surveys according to World Health Organization (WHO) guidelines. Thus, between the end of 2019 and the beginning of 2020, a large-scale serological survey of children 5–9 years was conducted in all health districts of Mali suspected of being endemic for onchocerciasis. The results of this investigation showed the seroprevalence thresholds measured by the OV16 rapid diagnostic test in the laboratory were below the WHO defined 2% level for success in all operational transmission zones, except for an operational transmission zone in the Kayes region, namely the KA05 zone, where there was 2.60% seropositivity to OV16.

## Introduction

Onchocerciasis is a parasitic disease due to *Onchocerca volvulus*, and in Mali it is transmitted by *Simulium damnosum s*.*l*.; it is endemic in five regions: Kayes, Sikasso, Koulikoro, Segou and Mopti [1,2]. Baseline prevalence assessments in humans took place in 34 health districts between 1971 and 1987 that showed a wide range of *O*. *volvulus* prevalence from 40% to 75% with community microfilarial loads as high as 33.94 microfilariae per skin snip. These assessments provided valuable data that allowed for the classification of the health districts/foci into hyper-endemic (12 health districts with a prevalence ≥ 60%), meso-endemic (15 health districts with a prevalence between ≥ 40% and <60%), and hypo-endemic (7 health districts with a prevalence <40%). Treatment strategies using vector control and/or ivermectin through mass drug administration (MDA) were developed and followed the standard prevalence classification guidelines for use. The onchocerciasis endemicity status of 40 health districts was unknown; however, these areas nevertheless benefited from ivermectin treatment being distributed as part of lymphatic filariasis (LF) MDA elimination interventions—LF being endemic in all the health districts of Mali [2].

Large scale onchocerciasis control started in 1975 with the Onchocerciasis Control Programme in West Africa (OCP). In Mali, the initial phase of the OCP covered the endemic areas of Sikasso, Eastern part of Koulikoro, Segou and Mopti with vector control as the key intervention. The control area was further extended in 1987 to Kayes region and the western part of Koulikoro region with ivermectin distribution as the principal intervention, supplemented with larviciding. At the closure of the OCP in 2002 and the subsequent decentralization of control activities to the Mali national program, ivermectin treatment was carried out initially by mobile teams, then by communities through community-based treatment with ivermectin (CBTI) and community-directed treatment with ivermectin (CDTI). In the remaining health districts where lymphatic filariasis was co-endemic with onchocerciasis, albendazole and ivermectin were administered to eliminate LF [3,4]. Overall, Mali has experienced over 40 years of vector control or ivermectin treatment for onchocerciasis.

Surveillance conducted since 2002 indicates that transmission may have been interrupted across most of the onchocerciasis programmatic area [5,6]. Additionally, studies in two endemic foci in the Bakoye River and the Falémé River (border of Mali and Senegal) using the bi-plex rapid tests for OV16/Wb123 showed a seroprevalence level below the 2% threshold in the pre-control hyperendemic foci [4,7].

After a review by the national onchocerciasis elimination expert committee, it was recommended that a Pre-Stop MDA survey be conducted in all endemic districts under treatment to determine whether a full Stop MDA survey should be carried out, in line with the World Health Organization (WHO) recommendations [8–11]. This current document presents the results of a Pre-Stop MDA survey following an update to the black fly breeding site map in the 20 endemic districts, and discusses the next steps towards the final decision of onchocerciasis elimination in Mali.

## Methods

### Ethical statement

The Ethical Committee of the Faculty of Medicine, Pharmacy and Odonto-Stomatology of Bamako, Mali approved the study (N°2019/ 166 /CE/FMOS/FAPH). National approval for the study was also granted for this study by the COMITE NATIONAL D’ETHIQUE POUR LASANTE ET LES SCIENCES DE LA VIE (CNESS)–Approval number: 2023-118/MSMS/CNESS. Community consent through the local leaders was obtained from each village and all the participants provided oral assent, and in addition oral consent was also obtained from guardians/parents for all children in the study prior to their participation.

### Pre-Stop MDA survey preparation

#### Assessment of onchocerciasis transmission potential

The assessment of each geographic area that might potentially support onchocerciasis transmission (otherwise known as “exclusion” mapping) was conducted during a workshop attended by national and international experts. The methodology consisted of looking at a detailed map of Mali and classifying geographic areas as onchocerciasis-free or as requiring to be surveyed based on the eco-climatic parameters of each area. For this exclusion mapping, all desert areas, rice-growing areas, areas with flat relief and areas where the hydrological network is very weak or non-existent were considered not to be adequate for onchocerciasis transmission. The ArcGIS version 10.8.1 (2020) computer tool was used to identify, region by region, the areas with river basins where prospection should be carried out. The choice of potential breeding sites was based on existing prevalence data, findings of prior epidemiological surveys of villages and vector collection sites, and eco-climatic information such as rainfall and hydrological network.

#### Updating information on black flies breeding sites and first-line villages

A workshop for trainers and data collectors was held in the Koulikoro region over ten days. The workshop had two phases: a theoretical phase that lasted two days and a field phase that lasted eight days. During the theoretical phase, presentations were given on the WHO Stop-MDA criteria and procedures based on available guidelines (11), the entomological pathways in onchocerciasis transmission, as well as the morphological characteristics of *S*. *damnosum*. The remainder of the training focused on effective implementation of activities to identify and map *Simulium* breeding sites. These include defining first-line villages, selection of the prospection team members; strategies for collaboration to address cross-border issues; the number of collection points needed per focus area; strategies for collecting at least 6000 flies per collection point; breeding site characteristics of interest, as well as the steps involved in conducting a breeding site survey. During the field phase, the workshop participants visited breeding sites to practice the lessons learned during the theoretical phase of the workshop.

Areas considered as potential *Simulium damnosum* s.l. breeding sites were surveyed to update the existing breeding site map. Knowledge of breeding sites within each of the operational transmission zones (OTZ) was then used to select first-line villages, or villages within a 5 km radius of a positive breeding site, for the epidemiological evaluation. A minimum of three and a maximum of 13 first-line villages were selected per OTZ. The number of first-line villages per OTZ was determined based on the size of the zone, recent prevalence data and time since last treatment.

### Implementation of the Pre-Stop MDA survey

#### Locations surveyed

**Fig 1:**
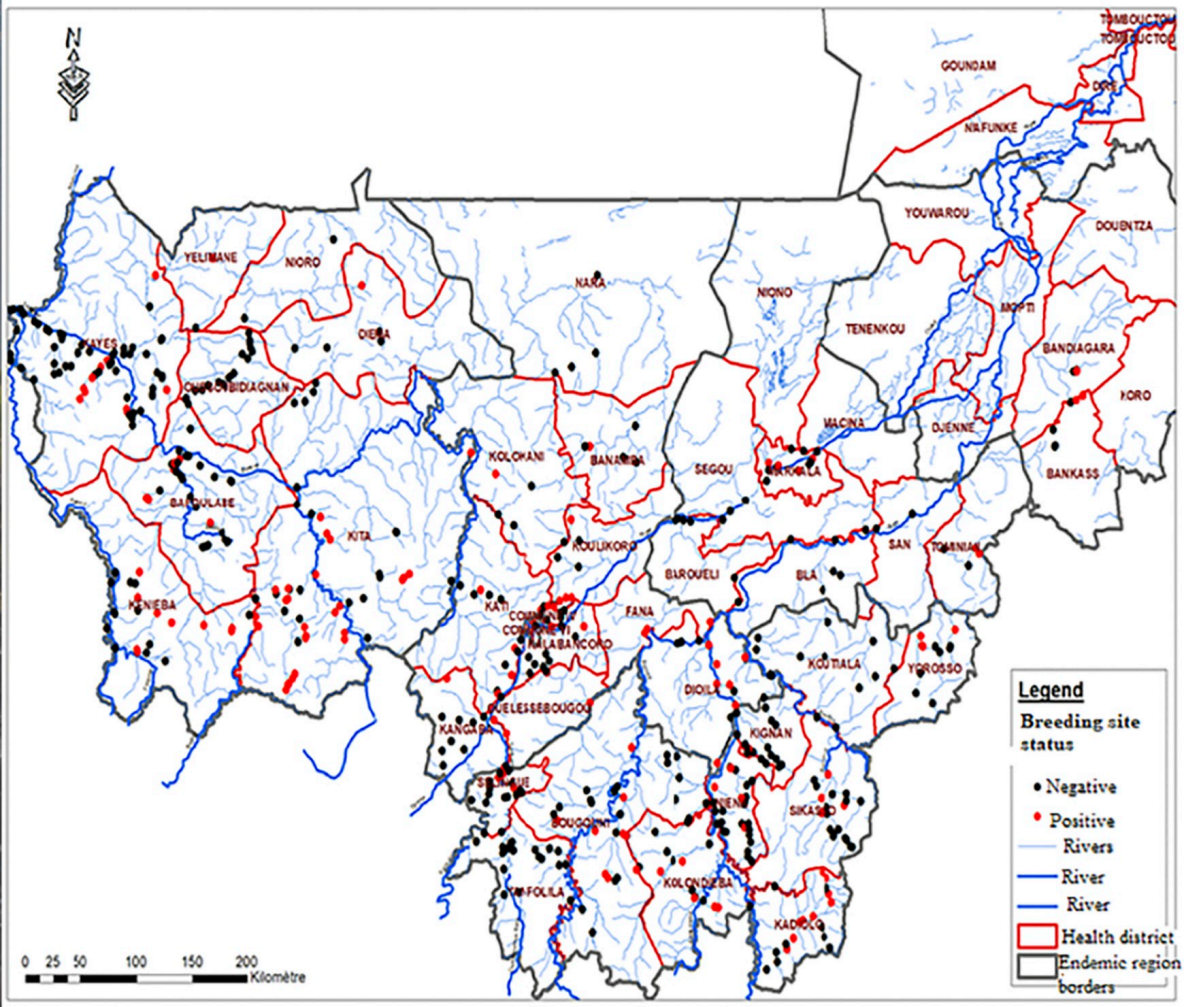
Map of Mali showing the positive and negative *Simulium* species breeding sites visited in the five endemic regions in Mali.

The Pre-Stop MDA survey was implemented as a cross-sectional study of all known endemic onchocerciasis foci based on OTZ in Kayes, Koulikoro, Sikasso, Ségou and Mopti regions. The survey included 1,022 eligible villages in 18 OTZs. The OTZs were demarcated based on ecological factors for vector breeding, movement of water in the river basins, and historical and current entomological and epidemiological data (Fig 1 shows the demarcation of the OTZs for each region). For the purposes of this survey, an OTZ was an eco-bioclimatic area within which the same vectors circulate [8–10].

**Fig 2:**
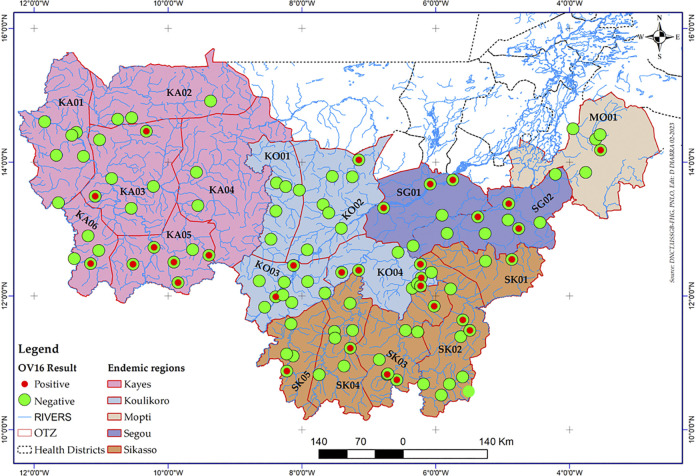
Spatial distribution of positive and negative children using OV16 serology and demarcation of operational transmission zones in the five regions in Mali.

A total of 18 OTZs were demarcated from the five endemic administrative regions. Kayes Region was then divided into six OTZs, Koulikoro Region into four OTZs, Sikasso Region has five OTZs, Segou Region has two OTZs, and Mopti Region has one OTZ (Fig 2).

Knowledge of breeding sites within each of the OTZs was then used to select first-line villages, or villages within a 5 km radius of a positive breeding site, in preparation for the epidemiological evaluation. One hundred (100) children aged 5 to 9 years (per OTS working documents–refs 8,9,10) were selected in each first-line village in the 18 OTZs (for details on the numbers of sites/villages selected per OTZ see Table 1). When the number of children in a selected village was less than the required 100, the remaining number of children were enrolled from the nearest villages. The targeted minimum sample size calculated was 9,700 children for all OTZ (8).

**Table 1:** OV16 seroprevalence in the five regions and 18 operational transmission zones.

Region	Focus	River	OTZ	# village surveyed	# of children tested	# Ov16 pos (%) /focus	% positive OTZ
**Kayes**	Kayes	Sénégal-Falémé	**KA 01**	3	300	0 (0)	0.00
	Kéniéba	Falémé		1	100	0 (0)	
	Bafoulabé	Sénégal		0	0	0 (0)	
	Yelimane	Sénégal	**KA 02**	1	100	0 (0)	0.00
	Kayes	Sénégal		0	NA	NA	
	Diema	Sénégal		1	100	0 (0)	
	Nioro	Sénégal		1	100	0 (0)	
	Kayes	Sénégal	**KA 03**	3	300	0 (0)	0.25
	Sagabary	Bagoé		0	NA	NA	
	Sefeto	Baoulé		0	NA	NA	
	Bafoulabé	Sénégal		4	400	1 (0.25)	
	Oussoubidiagna	Bafing-Bagoé		1	100	1 (1)	
	Diema	Sénégal	**KA 04**	0	NA	NA	0.00
	Sefeto	Sénégal		0	NA	NA	
	Kita	Baoulé-Bakoye		3	300	0	
	Sagabary	Baoulé-Bakoye	**KA 05**	4	400	7 (1.75)	2.60
	Bafoulabé	Sénégal		0	NA	NA	
	Kéniéba	Falémé-Bafing		1	100	6 (6)	
	Bafoulabé	Sénégal	**KA 06**	0	NA	NA	0.25
	Kéniéba	Falémé		4	400	1 (0.25)	
**Total 1**				**27**	**2700**	**16 (0.59)**	**0.59**
**Koulikoro**	Kolokani	Baoulé Ouest	**KO 01**	2	200	0 (0)	0.00
	Kati	Niger-Baoulé		1	100	0 (0)	
	Kolokani	Baoulé Ouest	**KO 02**	1	100	0 (0)	0.15
	Banaba	Niger		3	300	1 (0.33)	
	Koulikoro	Niger		5	500	0 (0)	
	Kati	Niger-Baoulé		4	400	1 (0.25)	
	Kalaban Coro	Niger		0	NA	NA	
	Bamako District	Niger		0	NA	NA	
	Moribabougou	Niger		0	NA	NA	
	Kati	Niger-Baoulé	**KO 03**	0	NA	NA	0.14
	Kangaba	Niger		3	300	1 (0.33)	
	Kalaban Coro	Niger		0	NA	NA	
	Ouelessebougou	Banifing		4	400	0 (0)	
	Kalaban Coro	Niger	**KO 04**	2	200	2 (1)	0.75
	Fana	Bani-Banifing-Baoulé-Bagoé		2	200	2 (1)	
	Ouelessebougou	Banifing		0	NA	NA	
	Dioila	Bani-Banifing-Baoulé-Bagoé		4	400	2 (0.05)	
**Total 2**				**31**	**3100**	**9 (0.29)**	**0.29**
**Sikasso**	Koutiala	Bagoé-Banifing	**SK 01**	4	400	3 (0.75)	1.4
	Yorosso	Bagoé		1	100	4 (4)	
	Koutiala	Bagoé-Banifing	**SK 02**	0	NA	NA	0.29
	Sikasso	Bagoé-Banifing		3	300	2 (0.67)	
	Kadiolo	Banifing		4	400	0 (0)	
	Kigan	Bagoé	**SK 03**	1	100	1 (1)	0.43
	Niéna	Bagoé		2	200	0 (0)	
	Kolondiéba	Bagoé		4	400	2 (0.5)	
	Bougouni	Baoulé-Ballé		0	NA	NA	
	Bougouni	Bagoé-Banifing	**SK 04**	3	300	1 (0.33)	0.2
	Yanfolila	Baoulé-Ballé		2	200	0 (0)	
	Bougouni	baoulé-Bagoé-Banifing	**SK 05**	0	NA	NA	0.33
	Yanfolila	Baoulé-Ballé		2	200	1 (0.5)	
	Sélingué	Sankarani		1	100	0 (0)	
**Total 3**				**27**	**2700**	**14 (0.52)**	**0.52**
**Segou**	Ségou	Niger	**SG 01**	1	100	0 (0)	0.75
	Baraouéli	Niger		1	100	1 (1)	
	Markala	Bani-Yame		2	200	2 (1)	
	Ségou	Niger	**SG 02**	0	NA	NA	0.4
	Bla	Bani-Yame		5	500	1 (0.2)	
	San	Bani-Yame		2	200	1 (0.5)	
	Tominian	Bani-Yame		3	300	2 (0.67)	
**Total 4**				**14**	**1400**	**7 (0.5)**	**0.5**
**Mopti**	Bandiagara	Bani-Yame	**MO 01**	3	300	0 (0)	0.2
	Koro	Bani-Yame		0	0	NA	
	Bankass	Bani-Yame		2	200	1 (0.5)	
**Total 5**				**5**	**500**	**1 (0.2)**	**0.2**

### Sample collection procedure for Pre-Stop MDA

#### Composition and training of the Pre-Stop MDA teams

The training for the serology surveys included two (2) days of theoretical training and one (1) day of field work. The theoretical training included sessions on community leaders’ information and sensitization, community mobilization for the planned activities, consent/assent obtained from parents or guardians, sample collection, dried blood samples (DBS) labeling and storage and laboratory processing of collected samples. During the field work component of the training, trainees underwent a mock village survey.

In total, there were eight (8) survey teams of four people each. Two team members were from the national level, one from the concerned administrative Region and one from the survey district level. The national level was represented by staff from the General Directorate of Health and Public Hygiene (DGSHP in French), the Neglected Tropical Diseases Research Unit of the Faculty of Medicine and OdontoStomatology (FMOS), the National Institute of Public Health (INSP in French). Field activities were supervised by experts from the national level in addition to Helen Keller International Mali office NTDs experts.

#### Blood sample collection

Survey teams worked in the pre-selected villages to collect data. In each village, a gathering area was identified by community leaders. Community health workers and schoolteachers then directed the children and their parents (or guardians) to the chosen location for sample collection.

A total of 360μl of blood was collected in six blood spots of 60 μl each on Whatman filter paper from each child [12]. The blood on the filter papers were dried at room temperature and stored individually in plastic bags labelled with the unique identification number of the child. Bagged samples were kept in isothermal boxes before being transferred to an available cold chain system in the closest appropriately equipped health facility. The DBS samples were stored at -20°C at the NTD Research Unit laboratory at the International Center of Excellence in Research of the Faculty of Medicine and OdontoStomatology in Bamako.

### Laboratory analyses

#### Serological testing using dried blood spots in the laboratory

Laboratory technicians were trained on sample reception, labeling, storage, and OV16 rapid diagnostic tests (RDT) performance for dried and frozen blood samples. The test used in the laboratory for the rapid diagnosis of onchocerciasis was the SD Bioline Biplex Onchocerciasis/LF IgG4 Rapid Test [2,13–15]. Standard laboratory procedures were followed for all steps in the laboratory from sample reception to the recording of results.

For extraction, 30μl of OV16 buffer (provided by the Nutman Parasitology Laboratory at the National Institutes of Health, USA) was placed in the first three wells (A1, B1 and C1) of a 96-well plate, and 60μl of OV16 RDT buffer in the remaining wells. One of the six Whatman filter paper ears containing the dried blood was cut and placed individually in a well containing the 60μl of OV16 RDT buffer; each ear was carefully pushed to the bottom of the well. To avoid contamination, the scissors used during the experiment were carefully cleaned with an alcohol-soaked absorbent cotton swab each time before being reused for the next blood sample. Then 10μl of the control solution was added to the 30 μl of OV16 buffer as follows: A1-Positive control OV16, B1-Negative control, with each control well thoroughly mixed with the 30μl OV16 buffer using careful pipetting. For each plate, a recording sheet was filled immediately after placing the controls and samples in the wells. Each plate was covered with aluminum foil and incubated overnight at 4°C in the refrigerator. The following day, 5μl of OV16 RDT buffer were added to the round well of the Biplex assay cassette. Then, 10μl of the eluted blood sample was added to the same round well for the sample testing. Four drops of test diluent supplied with the Biplex kit were added to the square well of each test cassette. Immediately after adding the four drops, the timer was set for 30 minutes. The sample identification number and the result and reading time were written on the SD Bioline Biplex Onchocerciasis/LF IgG4 Rapid Test. The identification number of each sample was recorded on the result sheet immediately after the reading time was set. Test results for OV16 were read 30 minutes later for each sample. Only OV16 results for onchocerciasis seroprevalence are reported here. We conducted quality control on our tested sample by retesting 10% of our samples by an expert senior Biologist from our laboratory and the results were distributed and discussed during our stakeholder meeting.

### Data analysis

Data were electronically collected in the field using an Open Data Kit (ODK) application. After processing the blood samples, the blood test results were entered into the Excel sheet downloaded from the ODK and then analyzed by transmission zone, and the seroprevalence of each OTZ calculated. For the interpretation of the results, the OTS recommendation of a 2% infection threshold was used; thus, any OTZ with a prevalence below 2% was considered to have passed the Pre-Stop MDA survey and was considered eligible for a full Stop MDA survey. Those OTZs above 2% were defined as needing to continue MDA.

## Results

Over the onchocerciasis endemic regions, the black fly breeding site locations were updated and mapped (Fig 1). There were a total of 18 OTZs in the five endemic administrative regions that had potential breeding sites: thus the Region of Kayes was organized into six OTZs, the region of Koulikoro into four OTZs, the region of Sikasso into five OTZs, the region of Segou into two OTZs and the region Mopti into one OTZ (Fig 2).

In a total of 104 first line villages surveyed, 47 individuals were tested positive for OV16 antibodies within the 10,400 tested children aged 5–9 years. In Kayes Region, in the KA05 OTZ, the OV16 seroprevalence was 2.60% (13/500) (Table 1). In the Sikasso Region, in OTZ SK01, despite a seroprevalence of 1.40%, there was a village with a seroprevalence of 4% (Table 1).

## Discussion

Mali has been addressing the control, and more recently the elimination of transmission, of onchocerciasis for many years, with either vector control or ivermectin distribution in the onchocerciasis endemic districts from 1974 to present. As of 2020, hyper- and meso-endemic onchocerciasis districts have received over 24 consecutive rounds of annual MDA with ivermectin; in addition, the hypo- and non-endemic districts received six (6) consecutive annual rounds of ivermectin as part of the treatment of the lymphatic filariasis endemicity that extends across all health districts in Mali.

A total of 18 OTZs, considered to be endemic for onchocerciasis in the regions of Kayes, Koulikoro, Sikasso, Segou and Mopti were assessed and reported here. Seventeen of the 18 OTZs were found to have a seroprevalences of less than 2%, and therefore are considered to have successfully passed their pre-stop MDA survey and qualified for the conducting full Stop MDA surveys according to the WHO guidelines and its onchocerciasis technical subgroup (OTS) meeting reports [8,10]. However, the OTZ KA05 in Kayes showed a seroprevalence of 2.60% (Table 1), with prevalence varying from 0% to 6% in the surveyed villages. The village with the highest prevalence is likely a transmission “hot spot”. Additionally, the OTZ SK01 in Sikasso had a seroprevalence of 1.40% but had one village with a seroprevalence of 4%. The villages in SK01 and KA05 OTZs require further investigation before a decision is made on whether to proceed with either a stop ivermectin MDA survey soon, or to begin implementing, alternative treatment strategies such as bi-annual ivermectin treatment, introducing MDA using moxidectin [16], or perhaps targeted vector control.

A sensitivity of 84% and a specificity 98–99% were reported for the SD Bioline Biplex Onchocerciasis/LF IgG4 Rapid Test [17]. In our study, we observed a high degree of agreement between pre-control endemicity level, patterns of seroprevalence during monitoring activities, and the Pre-Stop MDA findings [16]. Despite these seroprevalence findings, we recommend for the next step to consider, in addition to the number of rounds of MDA conducted, the achieved coverage rates for a more secure decision when conducting the stop MDA survey following the Pre-stop MDA survey. These are important factors that can guide the final decision on whether to proceed with the Stop MDA surveys in each OTZ in Mali. It should be noted that according to the third OTS report, the 2% serological threshold was regarded as being a temporary recommendation requiring more entomological data from the field to support or modify it.

Our experience in the studies described in this manuscript suggests that designing surveys and making decisions based on a “transmission zone” approach as opposed using geographical units based on districts, i.e., the “district” approach, is the best way for the national program to move forward to addressing the residual or “hot spot” areas where transmission persists. These areas may benefit from the use of alternative treatment strategies, such as, bi-annual ivermectin MDA or MDA using moxidectin, and targeted vector control. For the OTZs where no positive samples were detected, or where the seroprevalence is below the 2%, we suggest these be considered as having qualified for full stop MDA surveys.

## Conclusion

The analysis of blood samples from 5–9 years old from selected first line villages across the 18 OTZs using OV16 RDT in the laboratory showed that the majority of OTZs in Mali have reached the criterion that is needed to initiate full stop-treatment survey. However, it is likely that there are still a few residual hotspots of onchocerciasis transmission present; thus, there is a need to treat these residual onchocerciasis hotspots for a few more years and carry out various operational research studies before the making a final decision to move to full stop-treatment surveys in these areas. The current studies showed that evaluations based on OTZs is more closely related to the disease epidemiology and thus a true indication of the actual situation [18].

## Maps

The maps used in this manuscript were created by one of the authors of this manuscript, Dasine Diarra, using Qgis software version 3.22 (open access freeware) and the base shape files created by the author’s institution, the University of Social Sciences and Management of Bamako, Department of History and Geography, in collaboration with State services whose data are available for public use without restriction.
